# Assessing the potential for demographic restoration and assisted evolution to build climate resilience in coral reefs

**DOI:** 10.1002/eap.2650

**Published:** 2022-06-27

**Authors:** Lukas B. DeFilippo, Lisa C. McManus, Daniel E. Schindler, Malin L. Pinsky, Madhavi A. Colton, Helen E. Fox, Eden Tekwa, Stephen R. Palumbi, Timothy E. Essington, Michael M. Webster

**Affiliations:** ^1^ School of Aquatic and Fishery Sciences University of Washington Seattle Washington USA; ^2^ Department of Ecology, Evolution, and Natural Resources Rutgers University New Brunswick New Jersey USA; ^3^ Hawaiʻi Institute of Marine Biology University of Hawaiʻi at Manoa Kaneʻohe Hawaii USA; ^4^ Coral Reef Alliance Oakland California USA; ^5^ Department of Zoology University of British Columbia Vancouver British Columbia Canada; ^6^ Department of Biology, Hopkins Marine Station Stanford University Pacific Grove California USA; ^7^ Department of Environmental Studies New York University New York New York USA; ^8^ Present address: Resource Assessment and Conservation Engineering Division NOAA Alaska Fisheries Science Center Seattle Washington USA

**Keywords:** assisted evolution, climate change, coral bleaching, coral reef restoration, evolutionary rescue

## Abstract

Interest is growing in developing conservation strategies to restore and maintain coral reef ecosystems in the face of mounting anthropogenic stressors, particularly climate warming and associated mass bleaching events. One such approach is to propagate coral colonies *ex situ* and transplant them to degraded reef areas to augment habitat for reef‐dependent fauna, prevent colonization from spatial competitors, and enhance coral reproductive output. In addition to such “demographic restoration” efforts, manipulating the thermal tolerance of outplanted colonies through assisted relocation, selective breeding, or genetic engineering is being considered for enhancing rates of evolutionary adaptation to warming. Although research into such “assisted evolution” strategies has been growing, their expected performance remains unclear. We evaluated the potential outcomes of demographic restoration and assisted evolution in climate change scenarios using an eco‐evolutionary simulation model. We found that supplementing reefs with pre‐existing genotypes (demographic restoration) offers little climate resilience benefits unless input levels are large and maintained for centuries. Supplementation with thermally resistant colonies was successful at improving coral cover at lower input levels, but only if maintained for at least a century. Overall, we found that, although demographic restoration and assisted evolution have the potential to improve long‐term coral cover, both approaches had a limited impact in preventing severe declines under climate change scenarios. Conversely, with sufficient natural genetic variance and time, corals could readily adapt to warming temperatures, suggesting that restoration approaches focused on building genetic variance may outperform those based solely on introducing heat‐tolerant genotypes.

## INTRODUCTION

Coral reefs worldwide are threatened by a variety of local and global stressors, causing widespread declines in these important ecosystems (Pandolfi et al., 2003). One of the most conspicuous drivers of reef decline is ocean warming, which disrupts the association between coral animals and their algal endosymbionts in a process known as coral bleaching (Hughes et al., 2017). Bleaching reduces coral growth, calcification rates, fecundity, and resistance to disease, often resulting in complete colony mortality (Baird & Marshall, 2002; Bruno et al., 2007; Douglas, 2003; Harvey et al., 2018). Large‐scale bleaching events are becoming more frequent and are soon expected to become a regular phenomenon (Anthony, 2016; Frieler et al., 2013; Hughes et al., 2018; Van Hooidonk et al., 2016). As such, the fate of coral reefs appears bleak, with some studies predicting the widespread loss of these ecosystems by 2050 (Burke et al., 2011; Hoegh‐Guldberg et al., 2017; Van Hooidonk et al., 2016). Such an outcome would represent a precipitous reduction in the ocean's biodiversity (Knowlton, 2001) and a loss of valuable ecosystem services to hundreds of millions of people who depend on coral reefs for their livelihoods (Cinner, 2014; Costanza et al., 2014; Hoegh‐Guldberg et al., 2015). However, despite such grim predictions, there is growing recognition that corals exhibit natural variation in their vulnerability to thermal stress, suggesting the potential to adapt to rising temperatures through evolution (Barshis et al., 2013; Bay & Palumbi, 2014).

As bleaching events are becoming more common, several studies have noted that corals originating from warmer areas can withstand temperatures that bleach conspecifics (Oliver & Palumbi, 2009, 2011; Palumbi et al., 2014). Such variation in thermal tolerance can be due to differences in symbiont assemblages (Baker, 2001; Berkelmans & van Oppen, 2006), as well as physiological acclimatization arising from prior exposure to thermal stress (Brown et al., 2014; Howells et al., 2013; Palumbi et al., 2014). However, a substantial proportion of the variation observed in coral bleaching sensitivity is attributable to genetic factors, allowing for the possibility that populations could withstand climate change through adaptive evolution (Barshis et al., 2013; Bay & Palumbi, 2014; Palumbi et al., 2014; Walsworth et al., 2019). Although the apparent capacity for corals to adapt to higher temperatures is encouraging, it remains uncertain whether their realized rates of evolution will be sufficient to keep pace with climate change (Logan et al., 2014). It has been argued that as long‐lived, panmictic organisms, scleractinian corals may not be capable of rapid evolution (Hoegh‐Guldberg, 2014; Hoegh‐Guldberg et al., 2007, 2017). Other studies have suggested that corals may be able to evolve quickly enough to keep pace with moderate climate change, but would suffer adaptive collapse under more rapid warming (Bay et al., 2017; Matz et al., 2020; Quigley et al., 2019).

With the unprecedented threats that coral reefs face, scientists and managers are increasingly seeking active conservation interventions to restore and maintain these ecosystems (Anthony et al., 2017; Morikawa & Palumbi, 2019; van Oppen et al., 2015). One widely applied but costly (~US$350,000/ha; Bayraktarov et al., 2019) strategy is to propagate coral colonies *ex situ* and transplant them to reefs to restore degraded coral populations (but please refer to also Harrison, 2020; Harrison et al., 2021). Such “propagate‐and‐transplant” approaches could benefit corals in the face of climate change by enhancing population size (“demographic restoration”), or by accelerating adaptation to climate change through the deliberate selection, propagation, and distribution of genotypes with resistance to warmer temperatures (“assisted evolution”; Yetsko et al., 2020) as well. The latter may involve the translocation of thermotolerant colonies from warmer regions to introduce beneficial alleles to recipient populations (i.e., “assisted relocation”; Dixon et al., 2015) as well as selective breeding or genetic engineering of corals and their symbionts (Quigley et al., 2021; van Oppen et al., 2015, 2017; Voolstra et al., 2021).

Given the potential benefits and costs of demographic restoration and assisted evolution, there is an urgent need to evaluate the scope for success of these approaches as society considers strategies for conserving reefs in the face of climate change (van Oppen et al., 2015, 2017). Here, we use a generalized simulation framework to model the evolutionary and ecological dynamics of coral reef metacommunities in response to climate warming. Concurrent with warming, we simulated various forms of demographic restoration and assisted evolution to (1) identify the levels of supplementation and assisted evolution at which conservation benefits may be realized, (2) compare alternative spatial designs for coordinating restoration efforts to optimize conservation impact, and (3) identify the biological scenarios under which such active interventions are most likely to be needed.

## METHODS

### General overview

**FIGURE 1:**
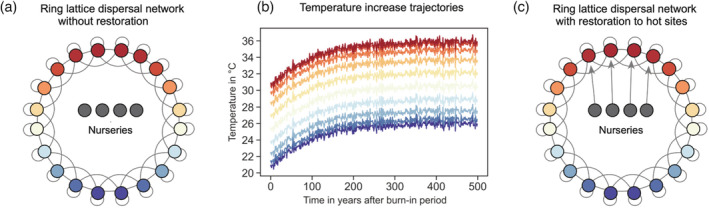
Schematics of the (a) ring lattice dispersal network without supplementation, the (b) temperature trajectories for each patch across the network during the temperature increase period, and (c) the dispersal network with supplementation to the four hottest sites. Each patch is connected to its four nearest neighbors as well as itself. Warmer and cooler colors are reefs that experience higher and lower temperatures, respectively. The optimal growing temperatures at the nursery reefs (*Z*_*a=s*_) were set depending on the degree of thermal trait enhancement.

To explore the effects of demographic restoration and assisted evolution, we simulated a coral reef metacommunity network consisting of 20 individual reef patches, each of which was populated by a coral subpopulation and a macroalgal competitor. Reef patches were organized across a temperature gradient of 3°C, with the hottest patches clustered on one side of the network and coldest patches on the other (Figure 1a). Reefs were connected through a ring lattice dispersal network in which coral subpopulations exchange larvae with their four nearest neighbors (i.e., the four subpopulations with the most similar thermal regimes to a given focal patch; McManus, Tekwa, et al., 2021). In addition to spatial thermal variation, we also simulated a network‐wide asymptotically increasing temperature trajectory with stochastic anomalies to simulate climate warming over the next century (Figure 1).

Concurrent with increasing temperatures, we tested various forms of demographic restoration and assisted evolution in which we varied the amount of new coral cover being added and the optimal growing temperature of the outplanted corals. Supplementation was simulated by creating additional “nursery patches” such that each target reef was connected to one nursery. We then tested a range of supplementation and assisted evolution levels by changing the fractional cover and mean thermal tolerance respectively at each nursery (Figure 1c).

### Eco‐evolutionary model

Our simulation framework is an extension of a previous model developed by Norberg et al. (2012) and modified by Walsworth et al. (2019), McManus, Forrest, et al. (2021) and McManus, Tekwa, et al. (2021) to simulate coral reef ecology under global change. On each reef patch, we simulated the dynamics of a single coral and macroalgal species that compete for space. We modeled both corals and macroalgae to include key community interactions that impact coral performance (Fung et al., 2011; McManus et al., 2019; Mumby et al., 2007). Additionally, there is the potential for alternative stable states of coral versus macroalgal dominance on reefs (Mumby et al., 2007) which may have important implications for the outcomes of restoration activities. Coral growth and mortality at a given reef patch were determined by local temperature relative to the coral subpopulation's evolvable optimum growing temperature. The change in coral cover through time at each subpopulation was determined according to:

(1) $$\frac{dC_{a}}{dt}=g_{C,a}C_{a}+l_{a}F_{a}+\frac{1}{2}V{\left.\frac{\partial^{2}g_{i}}{\partial z^{2}}\right|}_{z=z_{a}}C_{a},$$

[change in coral cover] = [population dynamics] + [larval immigration] + [genetic load]

where *C*_*a*_ is the fractional coral cover at reef *a*. Population dynamics of corals on reef *a* depended on their fitness (*g*_*C*,*a*_) and immigration, which was the product of the larval input rate (*l*_*a*_) and the amount of free space at that patch (*F*_*a*_). Each coral subpopulation at a given reef patch had an optimal growing temperature $\left(z_{a}\right)$ that could evolve over time through gene flow and selection. Evolutionary dynamics of the optimal growing temperature were specified as:

(2) $$\frac{dz_{a}}{dt}=\left(z_{i,a}-z_{a}\right)\left(\frac{l_{a}}{C_{a}}\right)F_{a}+q_{a}V{\left.\frac{\partial g_{C,a}}{\partial z}\right|}_{z=z_{a}},$$

[change in optimal growth temperature] = [gene flow] + [stabilizing selection]

where *z*_*i*,*a*_ is the population‐weighted mean trait value of larvae immigrating into patch *a*, *V* is the additive genetic variance and *q*_*a*_ reduced the strength of natural selection at very low coral cover.

Additive genetic variance (*V*) provided the raw material for evolution (Lande, 1976) but also affected population growth through a genetic load penalty $\left(\frac{1}{2}V\frac{\partial^{2}g_{C,a}}{\partial z^{2}}\right)$ (Kirkpatrick & Barton, 1997), which was either negative or zero. This was due to the concavity of the coral fitness function, which was affected by local temperature relative to a subpopulation's current optimum growing temperature. We assumed additive genetic variance to be constant. The effect of gene flow on the evolution of corals at a given patch was determined by the mean trait value (optimum growing temperature) of incoming larvae (*z*_*i*,*a*_) relative to the current trait value $z_{a}$ at that reef, as well as the larval input rate ($l_{a}$) and the amount of free space ($F_{a}$). Free space was defined as the habitat area that was not occupied by coral or macroalgae:

(3) $$F_{a}=1-\left(C_{a}+M_{a}\right).$$

As such, incoming larvae had a larger effect on the mean trait value if there was free space for settlement and if they represented a large fraction relative to the current coral cover. Selection on thermal optima was a function of additive genetic variance (*V*) and the fitness of a given trait value, with an additional term, $q_{a}$, which reduced selection at vanishingly low population sizes (Norberg et al., 2012).

The fitness (*g*_*C*,*a*_) of each coral subpopulation was determined by its growth (*r*_*C*,*a*_) and mortality (*m*_*C*,*a*_) rates, and interspecific competition that was encoded in an interaction matrix (α) where α_CM_ was the effect of macroalgae (*M*) on coral (*C*) and α_CC_ was the effect of coral on itself:

(4) $$g_{C,a}=r_{C,a}\left(1-\alpha_{\mathrm{CM}}M_{a}-\alpha_{\mathrm{CC}}C_{a}\right)-m_{C,a}.$$

where *M*_*a*_ is the percent area at reef patch a occupied by macroalgae. To test the sensitivity of the model to underlying assumptions of reef bistability (Dudgeon et al., 2010; Mumby et al., 2007), we specified two alternative parameterizations of the competition matrix (Appendix S1: Table S1) that allowed for alternative stable states or coexistence of corals and macroalgae. We explored both possible scenarios as it was unclear whether reef systems are best described by one or the other (Dudgeon et al. 2010; Mumby et al., 2007).

Growth (*r*_*C*,*a*_) and mortality (*m*_*C*,*a*_) of corals were specified as Gaussian and exponential functions, respectively, of local temperature ($T_{a}$) relative to a population's current optimal growing temperature $\left(z_{a}\right)$:

(5) $$r_{C,a}=\frac{r_{C,0}}{\sqrt{2\pi w^{2}}}\exp\left(\frac{-{\left(T_{a}-z_{a}\right)}^{2}}{w^{2}}\right)$$

(6) $$m_{C,a}=\left\{\begin{array}{ll} 0, & T_{a}\leq z_{a}\\ 1-\exp\left(\frac{-{\left(T_{a}-z_{a}\right)}^{2}}{w^{2}}\right), & T_{a}>z_{a} \end{array}\right.$$

where *r*_*C*,*0*_ is a coral growth scaling factor, and w is the width of coral thermal tolerance. Following Walsworth et al. (2019), an additional mortality cost was imposed when local temperature exceeded the thermal optimum ($T_{a}>z_{a}$).

Both population and evolutionary dynamics were influenced by dispersal among coral subpopulations. Following McManus et al. (2021), our model specified spatially explicit dispersal where larval settlement into a given patch increased with free habitat area, and a reef without free habitat area (*F* = 0) experienced a settlement rate of 0. Larval input, *l*_*a*_, was calculated from the connectivity matrix **D** in which $D_{\mathrm{ab}}$ was the probability of larvae from patch b reaching patch a, and the effective fecundity rate β:

(7) $$l_{a}=\beta \sum_{b}D_{\mathrm{ab}}C_{b}$$

Each reef patch was self‐connected and linked to four other patches (and if targeted for restoration, to a nursery patch as well, please refer to “*Simulating outplanting*”); all of these connections were of equal strength ($D_{\mathrm{ab}}=0.2$) and all patches were assumed to be equal in size.

In addition to affecting population dynamics, larval exchange among subpopulations or patches also contributed to the evolution of thermal optima across the reef network. Gene flow arising from dispersal was determined by the population‐weighted trait value of incoming larvae (*z*_*i*,*a*_) where the incoming trait values were calculated as:

(8) $$z_{i,a}=\frac{\beta \sum_{b}D_{\mathrm{ab}}N_{b}z_{b}}{l_{a}}$$

where $z_{b}$ is the average trait value of corals at patch b.

At vanishingly low coral cover (*C*_min_ = 10^−6^), the strength of directional selection was reduced through $q_{a}$ (Willi et al., 2006):

(9) $$q_{a}=\max\left(0,1-\frac{C_{\min}}{\max\left(C_{\min},2C_{a}\right)}\right)$$

The dynamics of macroalgae were modeled similarly to those of coral but were assumed to exhibit growth (*r*_*M*,*a*_) and mortality rates (*m*_*M*,*a*_) that were independent of temperature and set as constants. As such, instead of Equation (1), the population dynamics of macroalgae were specified as:

(10) $$\frac{dM_{a}}{dt}=g_{M,a}M_{a}$$

where:

(11) $$g_{M,a}=r_{M,0}\left(1-\alpha_{\mathrm{MC}}C_{a}-\alpha_{\mathrm{MM}}M_{a}\right)-m_{M,0},$$

and where *g*_*M*,*a*_ is the fitness of macroalgae, $\alpha_{\mathrm{MC}}$ is the competitive effect of corals on macroalgae, *C*_*a*_ is the percentage habitat area occupied by coral, *r*_*M*,*0*_ is the constant macroalgal growth rate, and *m*_*M*,*0*_ is the constant macroalgal mortality rate. Because macroalgae were primarily included as a competitor for coral, we assumed that macroalgae did not evolve and population dynamics were driven solely by local temperature‐independent growth and mortality, with no dispersal among reef patches.

### Simulating outplanting

In addition to the metacommunity with 20 reef patches, we simulated one nursery reef, *s*, for each reef patch that was targeted for demographic restoration and/or assisted evolution (Figure 1c). At each nursery, we defined a trait value (*Z*_*a=s*_) and replenished the fractional cover to the specified amount (*C*_*a=s*_) at every time step. As such, when there were *n* number of reefs targeted for restoration, there were a total of (20 + *n*) × (20 + *n*) elements in the connectivity matrix **D.** Outplanting of corals from the nurseries to target reefs was controlled by the connectivity matrix such that, **D**_*as*_ was either 1 or 0 depending on whether reef *a* was or was not the focal restoration site associated with that nursery reef, respectively. The larval input rate at each patch therefore includes recruitment from four neighboring patches, self‐recruitment, and input from the nursery reef, if applicable (Equation 7). If *a* was a target reef, the supplementation level each year for *a* was the product of its nursery reef's coral cover, the effective fecundity, and the current free space: β*C*_*a=s*_ *F*_*a*_. As in the natural larval recruitment process (second term on the right‐hand side of Equation 1), the effects of restoration were mediated by free space such that less free space led to lower overall recruitment. Because free space is a dynamic variable, we present supplementation levels based on the maximum potential supplementation rate, β*C*_*a=s*_, when *F*_*a*_ = 1 (*C*_*a*_ = 0 & *M*_*a*_ = 0).

### Climate conditions

Climate change was simulated as an asymptotically increasing temperature trend from a network‐wide average temperature of 27–32°C over ~200 years (Figure 1b). The magnitude of this increase was the same across all reef patches in the network, although the colder patches exhibited a lower starting temperature and therefore a lower asymptotic temperature compared with hot or intermediate patches. To account for interannual variation in temperature, we included network‐wide stochastic thermal anomalies overlaid on the directional temperature increase. Anomalies were normally distributed with a mean of zero and standard deviation of 0.3.

### Model simulations

Stochastic simulations were initiated for a “burn‐in” period of 1000 years in which the temperature remained stable but with annual anomalies. Fifty stochastic iterations were executed for each unique set of parameter combinations that we explored. Simulations began with corals occupying 25% of available habitat area at each reef patch with optimal growing temperatures that matched the average local temperature of their patch. Temperature increases began at the end of the burn‐in period, and the network dynamics were monitored for an additional 500 years.

We first explored how the vulnerability of corals to climate change varied across a range of values for genetic variance (V) and effective fecundity (β). No supplementation occurred in these scenarios, and we explored finer scale gradients of V and β. Variation in β represents an exploration of the overall larval immigration rate, which is equivalent to varying levels of $D_{\mathrm{ab}}$.

We then simulated various forms of demographic restoration and assisted evolution concurrent with climate change across the 500‐year monitoring period. We explored supplementation levels (β*C*_*a=s*_) of 0, 0.0000001 (1 × 10^−7^), 0.000001 (1 × 10^−6^), 0.00001 (1 × 10^−5^) and 0.0001 (1 × 10^−4^) target reef area year^−1^. Previous studies have indicated average reef area in the order of ~100 km^2^ in some regions (e.g., McManus et al., 2020; Schill et al., 2015). Assuming an average size of 100 km^2^ for each reef patch in the network, these values of β*C*_*a=s*_ amount to annual supplementation inputs of 0, 0.001, 0.01, 0.1, and 1 ha year^−1^ for 500 years. Existing propagate‐and‐transplant restoration efforts have been reported to have a median spatial extent of 0.01–0.02 ha and median duration of 1 year, although with some efforts up to 50 ha and larger efforts planned (Bayraktarov et al., 2019; Boström‐Einarsson et al., 2020). Outplanted corals' optimal growing temperature (*Z*_*a=s*_) was specified as 0, 1, 2, or 3°C greater than the target reef patch's current mean trait value. Because the optimal growing temperature of a given subpopulation could evolve over time, *Z*_*a=s*_ was re‐defined each year based on that reef's current trait value.

For each scenario, we assumed that 20% of the total network (four reef patches) was targeted for supplementation. We explored three possible spatial designs which targeted patches for restoration based on their temperature regime. The “hot strategy” focused restoration effort on the four hottest reefs (Figure 1c), representing a design that aimed to supplement sites that can serve as sources of warm‐adapted larvae for the entire network. The “cold strategy” supplemented the four coldest patches, representing a strategy of aiding sites that are predicted to fare well because of reduced thermal stress and favorable gene flow (Harrison, 2020; Matz et al., 2020; Norberg et al., 2012). Finally, the “random strategy” selected four reef patches at random, resulting in a portfolio that aimed to supplement a diversity of temperature regimes across the network.

All supplementation and assisted evolution scenarios were executed across levels of additive genetic variance values (V) ranging from no genetic variance (V=0) to low genetic variance (V=0.05) and moderate genetic variance (*V* = 0.1). We considered parameterizations of the competition matrix that produced either coexistence of corals and macroalgae or that produced alternative stable states (Appendix S1: Table S1; Tekwa et al., 2021). Unless otherwise specified, results described in the main text refer to simulations in which β = 0.01 and the competition matrix was parameterized for alternative states (Appendix S1: Table S1). Results including alternative parameterizations are presented in Appendix S1.

## RESULTS

### Biological scenarios (no supplementation)

**FIGURE 2:**
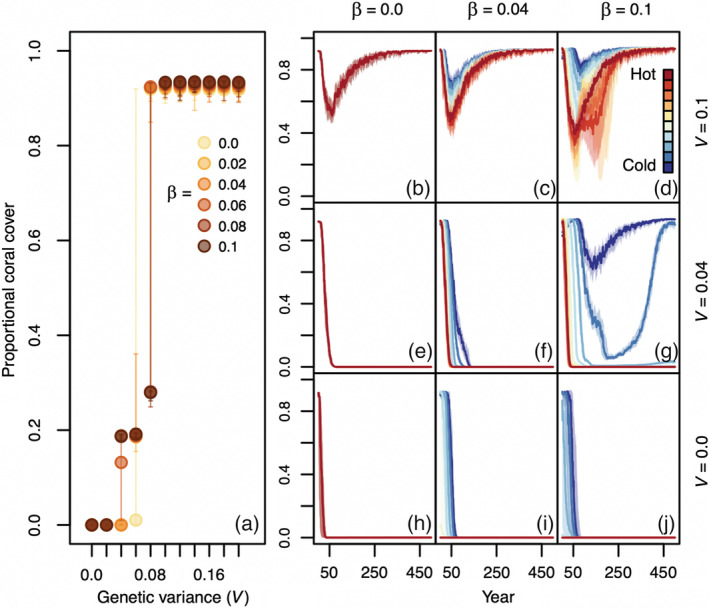
Coral responses to climate change across levels of coral effective fecundity/larval production (β) and genetic variance (*V*) without supplementation or assisted evolution. Circles in panel (a) show the median average network‐wide proportion of total habitat area occupied by coral at the end of simulated climate change trajectories across levels of genetic variance (*V*). Error bars represent 80% quantiles, and different colors correspond to values of β. Panels (b–j) show a simulated time series of coral cover across the reef network during climate change trajectories. Solid lines and shaded borders represent the median and 50% quantiles (across simulations), respectively, of coral cover over time at each patch within the network. Red and blue lines represent warmer and cooler patches, respectively. Simulations shown here assume a competition matrix parameterized for alternative stable states (i.e. coral‐ and macroalgal‐dominated regimes).

Of the biological variables considered, genetic variance had the largest effect on coral's resilience to climate change. In the absence of genetic variance (V=0), corals went extinct across the reef network in response to climate warming at all levels of effective fecundity (β) when supplementation did not occur (Figure 2h–j). Conversely, at moderate to high levels of genetic variance (i.e. V=0.1), corals at all patch types persisted, declining modestly between 50 and 250 years after warming began and then recovering to high levels of coral cover (>90%) by the end of the simulation (Figure 2b–d). Although coral subpopulations in all reef patches recovered by the end of the climate change projections at *V* = 0.1, the high‐temperature patches showed interim declines that were larger than in low‐temperature patches in some parameter combinations (Figure 2c,d).

The effects of increasing larval production (β) differed depending on the levels of genetic variance assumed. At low levels of genetic variance (V=0.04−0.06), higher effective fecundity (β = 0.08–0.1) more effectively maintained coral cover at the coldest patches (Figure 2g). At higher levels of genetic variance (*V* = 0.1), increasing larval production exacerbated interim declines in coral cover during climate change trajectories by impeding adaptation in the hot patches (Figure 2d). Results were qualitatively similar under a competition matrix parameterized for coexistence (Appendix S1: Figure S1).

### Demographic restoration and assisted evolution

**FIGURE 3:**
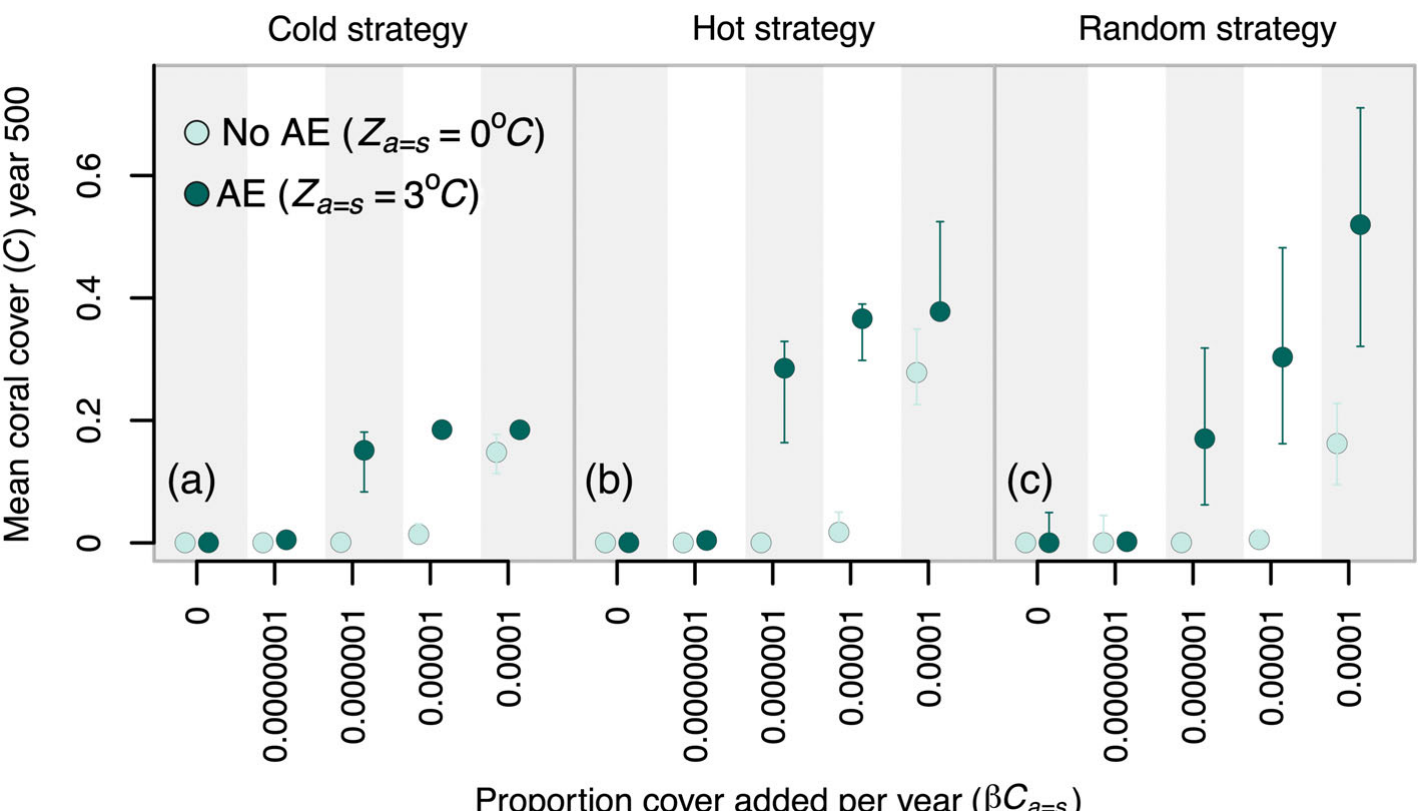
Effects of supplementation and assisted evolution on final (year 500) network‐wide average coral cover across spatial strategies. The *y*‐axis represents the median average network‐wide coral cover in year 500. Circles and 80% quantile bars are grouped on the *x*‐axis by annual maximum supplementation level (β*C*_*a=s*_), which represents the proportion of target reef patch area that was added to target sites each year for 500 years. For a standard network composed 100 km^2^ reefs, the values of β*C*_*a=s*_ on the *x*‐axis (0, 0.0000001, 0.000001, 0.00001, 0.0001) correspond to supplementation levels of 0, 0.001, 0.01, 0.1, 1 ha year^−1^. Light green circles represent restoration scenarios with no assisted evolution (AE) (trait enhancement of 0°C), although dark green circles represent scenarios with assisted evolution (trait enhancement of 3°C). Each panel represents a different spatial design of supplementation efforts, where panel (a) shows a strategy targeting the coldest patches, panel (b) shows a strategy targeting the hottest patches and panel (c) shows a strategy targeting randomly selected patches. Simulations shown here assume a competition matrix parameterized for alternative stable states between corals and macroalgae with V=0.05 and β = 0.01.

**FIGURE 4:**
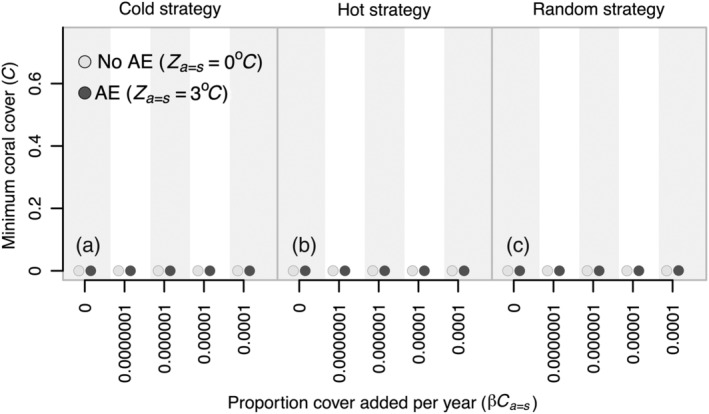
(a–c) Effects of the supplementation and assisted‐evolution efforts on minimum coral cover. Details of this figure are identical to those of Figure 3 except that plotted values represent the minimum proportional amount of coral cover that occurred during the 500 year simulations across different spatial designs and varying levels of supplementation and assisted evolution. Please refer to Appendix S1: Figures S5–S10 for the full trajectories

There was generally no long‐term benefit for the full reef network from supplementation when the thermal tolerance of outplanted colonies was not enhanced (i.e., demographic restoration only, *Z*_*a=s*_ = 0), except at the highest annual input levels considered (β*C*_*a=s*_ = 0.0001 year^−1^) (Figure 3; Appendix S1: Figure S2). Assisted evolution (*Z*_*a=s*_ > 0) also did not improve coral cover when annual supplementation was less than 0.000001 year^−1^ of target reef area (<0.01 ha year^−1^ assuming a 100 km^2^ reef) (Figure 3; Appendix S1: Figure S2, but please refer to Appendix S1: Figure S3). Conversely, supplementation rates of β*C*_*a=s*_ *≥* 0.000001 year^−1^ coupled with increased thermal tolerance (i.e., assisted evolution, *Z*_*a=s*_ > 0) in outplanted colonies lead to substantial improvements in final coral cover (e.g. from 0% up to 50% final coral cover in year 500; Figure 3). In some cases, comparable improvements could be realized at lower supplementation levels (0.0000001 year^−1^; 0.001 ha year^−1^ assuming a 100 km^2^ reef) under parameterizations of the competition matrix associated with coexistence of corals and macroalgae rather than alternative stable states (Appendix S1: Figure S3). Although sufficient levels of trait enhancement and supplementation could produce benefits in the level of coral cover present at the end of the simulations (year 500), no amount of demographic restoration or assisted evolution that we considered prevented the interim declines in coral cover to <1% during climate change trajectories (Figure 4; Appendix S1: Figures S4–S10). Even in parameter space where climate change did not lead to such drastic reductions in coral cover in the absence of interventions, demographic restoration and assisted evolution had little effect on improving the minimum amount of coral cover that occurred during climate change trajectories (Appendix S1: Figure S4). However, supplementation tended to primarily influence the degree to which corals recovered after these interim declines (Appendix S1: Figures S5–S10).

Demographic restoration and assisted evolution only produced benefits when genetic variance was nonexistent (V=0) or low (V=0.05) (Appendix S1: Figures S2, S3). At moderate levels of genetic variance (V=0.1), coral cover exceeded 90% by the end of climate change projections across all scenarios explored, and supplementation or assisted evolution produced no discernible benefits (Appendix S1: Figures S2, S3). No combination of supplementation level and trait enhancement that we considered led to levels of final coral cover equivalent to scenarios in which *V* = 0.1 (Appendix S1: Figures S2, S3). Similarly, interim declines in coral cover for scenarios where *V* = 0.1 were substantially smaller compared with scenarios of low (*V* = 0.05) or no (*V* = 0) genetic variance, even with high levels of supplementation and trait enhancement (Appendix S1: Figures S5–S10).

**FIGURE 5:**
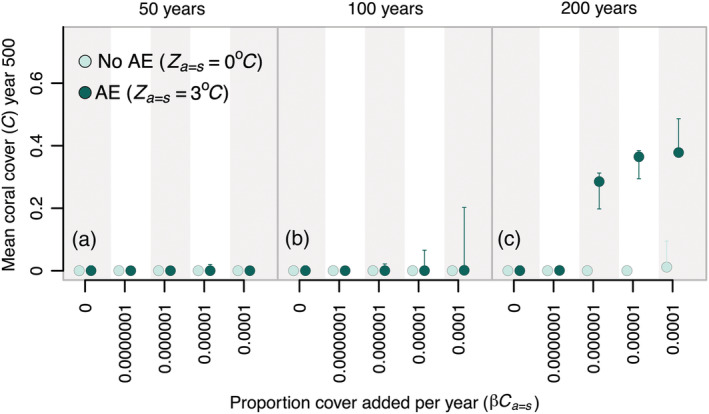
Effects of the duration of supplementation and assisted‐evolution efforts on final average coral cover. Details of this figure are identical to those of Figure 3 except that each panel represents a different duration for which supplementation was sustained ranging from 50 years (panel a) up to 100 years (panel b) and 200 years (panel c). Results shown here are from a supplementation strategy targeting hot reefs.

Results described so far have assumed that supplementation occurs annually for 500 years. Simulations that included supplementation over shorter timeframes showed that comparable outcomes could be achieved by supplementing for only the first 200 years, but that performance was distinctly reduced for supplementation periods of 50 or 100 years (Figure 5; Appendix S1: Figure S11). For much of the parameter space that we explored, supplementation that was only sustained for 50–100 years had little positive impact on final coral cover (Figure 5; but please refer to Appendix S1: Figure S11). Similarly, although the results described so far have used performance metrics based on a 500‐year time horizon (i.e. mean coral cover at year 500, minimum coral cover across 500 years), we also evaluated outcomes measured over shorter time scales. No level of annual supplementation and trait enhancement that we considered produced any discernible benefit in coral cover within the first 200 years of the simulation, and there were only slight improvements after 300 years (Appendix S1: Figures S12–S16).

At the network‐wide level, supplementation strategies that focused on the most thermally vulnerable metapopulation segments (hot patches) or selected a wide range of sites (random patches) performed comparably with one another and outperformed strategies that targeted thermal refugia (cold patches) (Figure 3). For supplementation levels of 0.000001 to 0.0001 year^−1^ and trait enhancement of 3°C, the median network‐wide average coral cover among simulations ranged from 0.15 to 0.18 (proportion of total reef area) for the cold strategy, 0.29 to 0.38 for the hot strategy, and 0.17 to 0.52 for the random strategy (Figure 3). Although the strategy targeting cold patches was generally effective at promoting coral cover at these sites (Appendix S1: Figures S7, S8), the benefits did not spill over into other patches, resulting in reduced network‐level performance. In contrast, strategies targeting hot patches and a random set of patches tended to be successful both at promoting coral cover at the target patches and at neighboring sites (Appendix S1: Figures S5, S6, S9, S10). Importantly, different spatial designs did not affect the supplementation and trait enhancement thresholds required to produce benefits.

## DISCUSSION

We used a simulation model to explore how supplementation might promote resilience to climate change on coral reefs via augmenting population size alone (demographic restoration) as well as introducing heat‐tolerant genotypes (assisted evolution). In most model scenarios without supplementation, corals subjected to climate warming were extirpated unless existing genetic variance was sufficiently high (i.e., *V* = 0.1). In simulations in which supplementation did occur, it offered little benefit for coral reefs faced with climate warming unless the thermal tolerance of outplanted colonies was also enhanced (i.e. demographic restoration had little benefit). This result is not necessarily surprising; when a population is maladapted to a changing environment and experiencing high mortality, any equally maladapted individuals added to the population will presumably die as well. In some cases, adding such genetically maladapted individuals could be deleterious and delay evolutionary rescue (Carlson et al., 2014). We avoided these effects in our simulations because the thermal tolerance of outplanted corals tracked that of the target population. Nonetheless, our results suggested that demographic restoration is unlikely to produce climate resilience benefits for corals, except at large and sustained effort levels. However, supplementation of coral populations may provide important benefits towards recovery from other stressors (e.g., mining, pollution, sedimentation) not considered here.

Increasing the thermal tolerance of outplanted colonies (assisted evolution) reduced the input levels necessary to achieve long‐term benefits. However, even the highest levels of assisted evolution that we considered generally failed to prevent severe and protracted reductions in coral cover during simulated climate change. Although assisted evolution facilitated the recovery of corals after these interim declines in some cases, such benefits were usually only achieved at supplementation rates of at least 0.000001 year^−1^ sustained over ≥100–200 years. The feasibility of such supplementation levels naturally depends on the size of the target reefs, which should be a consideration in determining a site's suitability for assisted evolution. For instance, supplementing one‐fifth of the total reef network (as was done in our simulations) at a rate of 0.000001 year^−1^ would equate to adding roughly 6.89 ha year^−1^ of thermally enhanced corals to the Great Barrier Reef, (http://www.gbrmpa.gov.au), 0.04 ha year^−1^ for Hawai‘i's Maro Reef (https://www.papahanaumokuakea.gov) and 0.02 ha year^−1^ for the Belize Barrier Reef Reserve System (https://whc.unesco.org/en/list/764/). To sustain these efforts over 100–200 years would amount to a total effort of 689–1378 ha for the Great Barrier Reef, 4–8 ha for Maro reef, and 2–4 ha for the Belize Barrier Reef Reserve System. In contrast, reviews of documented propagate‐and‐transplant restoration efforts have indicated a much smaller median spatial extent of 0.01–0.02 ha sustained for only 1 year (Bayraktarov et al., 2019; Boström‐Einarsson et al., 2020; Hein et al., 2017). At the reported median cost of US$351,661 ha^−1^ for propagate‐and‐transplant restoration (Bayraktarov et al., 2019), the cost of supplementing the Great Barrier Reef at this level would be US$2.4 M per year for 100–200 years, not accounting for the costs of developing the thermally enhanced corals or mortality of outplanted colonies. Purely demographic restoration at a level of 0.0001 per year (689 ha year^−1^) would cost in the order of US$240 M per year, roughly 4% of the economic contribution of the Great Barrier Reef to the Australian economy (https://www.barrierreef.org/the-reef/the-value). For smaller reefs (e.g. atolls) the necessary input thresholds are naturally more achievable. For instance, a 20 km^2^, reef would only require 0.0004 ha year^−1^ of thermally enhanced corals (0.04–0.08 ha total across 100–200 years). These estimates are purely illustrative because they assume that the entire reported spatial extent for a given reef system is composed of a viable coral habitat that could be targeted for supplementation. Additionally, these costs and effort calculations do not account for outplanting mortality or additional expenses that may be associated with assisted evolution beyond those of demographic restoration (although transplanting corals without propagation [e.g., assisted relocation] has a lower median cost of US$218,305/ha; Bayraktarov et al., 2019). Nonetheless, our simulations showed that even at these supplementation levels, benefits were not realized within the first 200 years of supplementation.

With regard to spatial designs for assisted evolution efforts, we found a comparable performance between approaches that targeted the warmest, most thermally vulnerable subpopulations (hot strategy) and those focused on a portfolio of patches (random strategy). Targeting thermal refugia (cold strategy) performed relatively poorly, similar to results for conservation strategies using marine protected areas (Walsworth et al., 2019). Although cold patches are likely to be the most protected from temperature extremes during climate change, their populations also have the lowest thermal optima. Because trait selection of new corals was defined relative to the mean value at target patches, the trait values of thermally enhanced corals added to the cold reefs were still relatively low compared with the thermal regimes of other patches in the reef network. Although they experienced local benefits from supplementation and assisted evolution, cold patches did not serve as a source of beneficial thermotolerant larvae to other patches. As such, supplementing these reefs offered little in the way of network‐level conservation benefits. Conversely, the hottest patches in the network could serve as a source of thermotolerant larvae to the rest of the reef network, such that targeting and maintaining these patches produced stronger network‐level benefits. Similarly, targeting a mixture of reef patches dispersed the benefits of assisted evolution across a broader area of the reef metacommunity, promoting higher overall coral cover across the network. Consequently, restoration activities may benefit from distributing inputs across a diverse range of habitats, including the most thermally stressed, rather than focusing narrowly on predicted climate refugia.

Our simulations did not consider several factors that could impact the outcomes of demographic restoration and assisted evolution in practice. Notably, we did not consider possible genetic tradeoffs between thermotolerance and other fitness‐related traits such as growth, cold‐tolerance, susceptibility to disease and sedimentation, and sensitivity to ocean acidification (D'Angelo et al., 2015; Hume et al., 2016). We also did not model potential interactions between assisted evolution and standing genetic variance. In reality, supplementing reefs with large quantities of a narrow range of thermotolerant genets may erode the genetic variance of a target population and reduce its adaptive capacity (Baskett et al., 2009; Sgrò et al., 2011; Shearer et al., 2009). Furthermore, our simulations specified supplementation levels in terms of successfully transplanted corals that survive to reproduce. Outplanted corals can suffer high early mortality (~35%–40%) due to the stresses of translocation (Boström‐Einarsson et al., 2020; Casey et al., 2015), such that in situ supplementation levels would need to be higher than those that we specified to offset losses. However, we note that the realized supplementation levels in many years of our simulations were substantially (e.g., ~60%–70%) lower than the specified maximum values (β*C*_*a=s*_) due to the scaling of inputs by the amount of free space on target reefs (*F*_*a*_), which was often limited due to proliferation of macroalgae (Appendix S1: Figure S17). Similarly, our analysis assumed no impact of climate change on macroalgal dynamics. Although the realized impact of climate change on macroalgae remains equivocal (Ji & Gao, 2021), any such effects could have important implications for spatial competition on reefs and the potential outcomes of supplementation. Our analyses also assumed that all reefs were homogeneous except for local temperature and ignored the potential impacts of other stressors beyond warming, such as ocean acidification, eutrophication, overfishing, disease, sedimentation, and pollution (Harvey et al., 2018; Knowlton, 2001; Pandolfi et al., 2011). In reality, individual reefs differ across several axes such as habitat quality and community composition that would impact local growth rates and carrying capacity. Furthermore, the presence of multiple stressors would probably complicate the selection of advantageous genotypes, as corals that are resilient to warming may be more vulnerable to other impacts (but please refer to Wright et al., 2019).

Demographic restoration and assisted evolution carry risks that we did not consider in our simulations (Baums, 2008). Knowledge of the genetic architecture underlying thermal resistance in corals is limited, and selection for a single trait could undermine resilience to other stressors via unforeseen genetic correlations (Ladd et al., 2017; Muller et al., 2018). Artificial selection for a single trait also risks creating genetic bottlenecks that erode adaptive capacity (Baums et al., 2019; Sgrò et al., 2011; Shearer et al., 2009). Moreover, selecting genotypes that are better suited to future conditions requires a level of certainty about ecosystem‐level responses to warming that may be unrealistic (Schindler & Hilborn, 2015; Webster et al., 2017). However, supplementation activities may also offer other conservation advantages that our analysis did not consider, such as community engagement (Hein et al., 2017; Kittinger et al., 2016), restoration of reefs degraded from non‐climate stressors, and a means to augment genetic diversity (Baums et al., 2019). In cases in which coral populations have been reduced to precipitously low levels, supplementation may help overcome demographic and genetic bottlenecks and stimulate natural adaptation by facilitating sexual reproduction. Although our simulations assumed a starting coral cover of 25%, many natural reefs are already below this level and therefore may benefit from population augmentation to increase reproductive potential and genetic diversity.

Our results support previous findings that the amount of genetic variance underlying thermal tolerance is key to shaping corals' ability to keep pace with climate change (Baskett et al., 2009; Walsworth et al., 2019). In the absence of genetic variance, corals were rapidly extirpated in response to warming. Conversely, at moderate levels of genetic variance (*V* = 0.1) corals could successfully adapt to warming and persist at high levels. Although empirical estimates of genetic variance underlying thermal tolerance in corals are scarce, there is evidence to suggest that the range considered in this study, as well as higher values, are plausible (Cornwell et al., 2020; Palumbi et al., 2014). Cornwell et al. (2020) reported that the phenotypic variance in thermal tolerance was 0.56 for *Acropora hyacinthus*, and Palumbi et al. (2014) estimated the narrow sense heritability (*h* ^2^) of this trait as 0.5 for *A. hyacinthus*, although Dziedzic et al. (2019) reported *h* ^2^ of 0.58 for *Orbicella faveolata*. Genetic variance of a trait is defined as the product of its phenotypic variance and narrow sense heritability, resulting in levels of *V* from 0.28 to 0.32 for this range of *h* ^2^. Additional studies that estimate levels of genetic variance in coral populations across a range of species and locations are likely to be particularly useful for predicting adaptive responses and prioritizing sites for interventions.

Although we tested different combinations of parameter values to assess the robustness of our simulation results (Appendix S1: Figures S1–S16), it is important to note that our model was not developed to represent a specific reef system (McManus et al., 2020; Walsworth et al., 2019). Consequently, the supplementation and trait enhancement values that we have reported should not be viewed as precise targets to guide management decision‐making. However, our results serve to illustrate the magnitude of effort needed to produce benefits, and the general timescales over which benefits may be realized. Applying the supplementation levels tested in our study to real‐world reef systems assumes that input requirements scale linearly with the size of target reef systems, which may not necessarily be accurate due to scale‐dependent variation in connectivity patterns and other ecological processes (Levin, 1992). In line with our results, however, Condie et al. (2021) evaluated the outcomes of assisted evolution in a simulation model parameterized and validated for the Great Barrier Reef and found little benefit from this approach alone over a 50‐year time horizon, despite using a higher level of annual supplementation (10 ha year^−1^ to each target reef) than those considered in this study. Quigley et al. (2019) reached a more optimistic conclusion about the potential for assisted evolution to improve outcomes for the Great Barrier Reef, although the supplementation levels that they explored appeared to be substantially greater than those we consider feasible here.

Although we found that assisted evolution could be beneficial when maintained at sufficiently high levels, inputs typical of existing restoration efforts failed to produce ecologically significant benefits in our simulations (Bayraktarov et al., 2016, 2019; Boström‐Einarsson et al., 2020; Hein et al., 2017). Furthermore, the high costs of supplementation suggest that a sustained upscaling in effort may not be readily attainable. Costs are likely to be even higher for assisted evolution that involves additional labor‐intensive steps such as sourcing and transporting heat‐tolerant corals or selectively breeding or engineering colonies in captivity. However, we note the possibility of decreasing restoration costs with increasing scale, or possible improvements in restoration technology (e.g., Harrison et al., 2021), which may reduce the effort levels required for effective supplementation. Although the costs and input levels that we estimate may be achievable for some ecosystems, the performance of supplementation and assisted evolution by themselves may not warrant exclusive investment in these strategies. We found that large assisted evolution inputs could facilitate the recovery of coral reefs from climate change impacts over extended time horizons (500 years), no benefits were realized within the time periods typically relevant to managers and stakeholders (i.e., ≤50–100 years), and efforts needed to be sustained over ≥100–200 years to be effective. Moreover, even at these effort levels, prolonged reductions in coral cover during climate change trajectories still occurred. As such, our results indicated that, although supplementation and assisted evolution offer benefits, by themselves these strategies may not be sufficient to mitigate climate change impacts on coral reefs. However, we did not evaluate the performance of supplementation in concert with other conservation measures (e.g., Condie et al., 2021). Supplementation in conjunction with activities such as reducing sewage runoff and fishing pressure on herbivores to control algal growth may be more effective than supplementation alone.

We found that in scenarios in which corals had sufficient genetic variance to keep pace with climate change, coral cover declined only modestly as the climate warmed, and recovered faster than could be achieved through supplementation and assisted evolution. As such, our results suggest that assisted evolution efforts cannot offer comparable benefits with the presence of robust adaptive capacity. This finding lends support to the restoration framework put forth by Baums et al. (2019), which prioritizes maintaining and building genetic variance rather than focusing solely on the propagation and distribution of thermally tolerant genotypes. Although Baums et al. (2019) advocated for including thermotolerant colonies in supplementation programs, they also recommend including a broad range of phenotypes and establishing sexually reproducing populations to promote genetic variance and adaptive potential. Such an approach minimizes the risks from potential genetic tradeoffs associated with thermal tolerance, offers resilience to a broader range of stressors, and has the potential to enhance standing genetic variance, which appears to be the most important factor in determining the ability of coral populations to withstand climate change. As such, supplementation efforts that focus on promoting adaptive potential by building genetic variance may outperform those aimed solely at introducing thermotolerant colonies. Other conservation measures beyond supplementation may also be useful in protecting corals' genetic variance, and concurrently pursuing a portfolio of intervention strategies to mitigate multiple impacts may forestall extirpation and allow populations with sufficient genetic variance time to adapt to rising temperatures (Condie et al., 2021).

## CONFLICT OF INTEREST

The authors declare no conflict of interest.

## Supporting information


Appendix S1

## Data Availability

Simulation model codes (DeFilippo & McManus, 2022) are available in Zenodo at https://doi.org/10.5281/zenodo.6353666.
