# Supplementary material for: Assessing the potential for demographic restoration and assisted evolution to build climate resilience in coral reefs
Source: Ecol Appl. 2022 Jun 27;32(7):e2650. doi: 10.1002/eap.2650 (PMC9788104; doi:10.1002/eap.2650)
Supplement: Supplementary file 1 — Appendix S1 [file EAP-32-e2650-s001.pdf]

Ecological Applications

Appendix S1. Assessing the potential for demographic restoration and assisted evolution to build climate resilience in coral reefs

Lukas B. DeFilippo, Lisa C. McManus, Daniel E. Schindler, Malin L. Pinsky, Madhavi A. Colton, Helen E. Fox, Edward W. Tekwa, Stephen R. Palumbi, Timothy E. Essington, Michael M. Webster

Table S1. Parameter definitions and values. Different sets of parentheses denote ranges used for different phases of the analysis.

Symbol	Parameter	Values
V	Additive genetic variance	(0, 0.05, 0.1)<br><br>(0.02, 0.04, 0.06, 0.08, 0.1, 0.12, 0.14, 0.16, 0.18, 0.2)
β	Effective fecundity	(0.01, 0.1)<br>(0.02, 0.04, 0.06, 0.08, 0.1)
α_{CM}	Effect of macroalgae on coral	Alternative stable states: 1.2 Coexistence: 1
α_{MC}	Effect of coral on macroalgae	Alternative stable states: 1 Coexistence: 0.8
$r_{C,0}$	Scaling factor for coral growth rate	1.5
$m_{0,m}$	Macroalgal mortality	0.15
$r_{M,0}$	Scaling factor for macroalgal growth rate	1.0
w	Thermal tolerance breadth	1.5

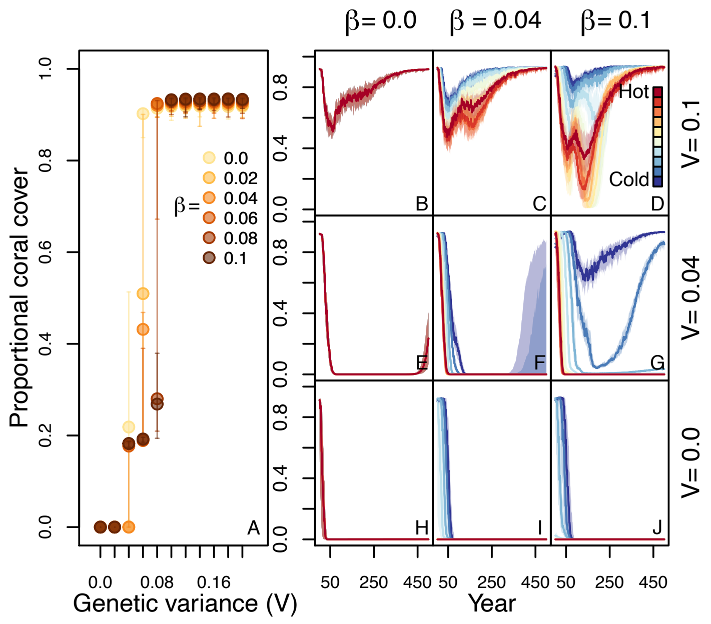

Figure S1. Coral vulnerability to climate change across levels of dispersal and genetic variance. The details of this figure are identical to Figure 2 except that the competition matrix is parameterized for coexistence between coral and macroalgae rather than for alternative stable states.

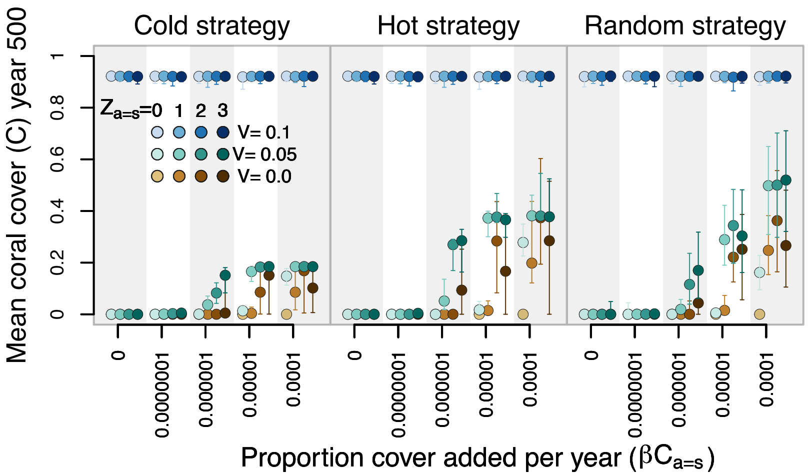

Figure S2. Effects of supplementation and assisted evolution on mean final coral cover. The details of this figure are similar to those of figure 3 in the main text but expanded to include the full range of parameter values considered. The y axis is the average across the reef network in the final year (year 500) of the simulation after 500 years of annual supplementation at the levels indicated on the x axis. Circles represent the median average network-wide coral cover at the end of simulated climate change, and error bars represent 80% quantiles among simulations. Blue, teal and brown circles represent scenarios of moderate ($V = 0.1$), low ($V = 0.05$) and no ($V = 0$) genetic variance respectively. For a given color series, the shade represents the trait enhancement of corals being added to the reefs through supplementation, with the lightest shade representing a trait enhancement of 0 (indicating that the thermal optimum of new corals was identical to the target reef average), and the darkest shade corresponding to a trait enhancement of 3 degrees (indicating that the thermal optimum of new corals was 3 degrees greater than the target reef average). Circles are grouped by annual supplementation amount (x-axis), which represents the proportion of target reef habitat area that was added to target reefs each year through supplementation. Coral fecundity (β) is equal to 0.01 in all panels. Each panel represents a different spatial design for supplementation efforts, where the first panel represents a strategy targeting the coldest patches, the second panel represents a strategy targeting the hottest patches and the third panel represents a strategy targeting random. Simulations shown here assume a competition matrix parameterized for alternative stable states between corals and macroalgae.

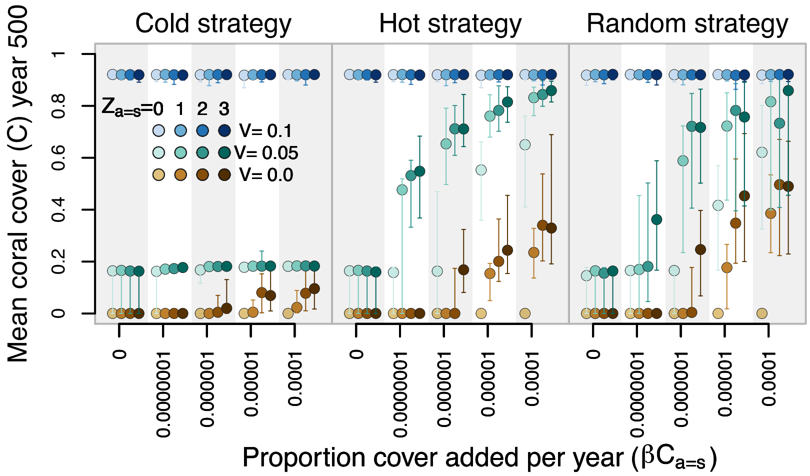

Figure S3. Effects of supplementation and assisted evolution on mean final coral cover in simulations parameterized for coexistence. The details of this figure are identical to those of Figure S2 but parameterized for coexistence between corals and macroalgae versus alternative stable states (Table S1).

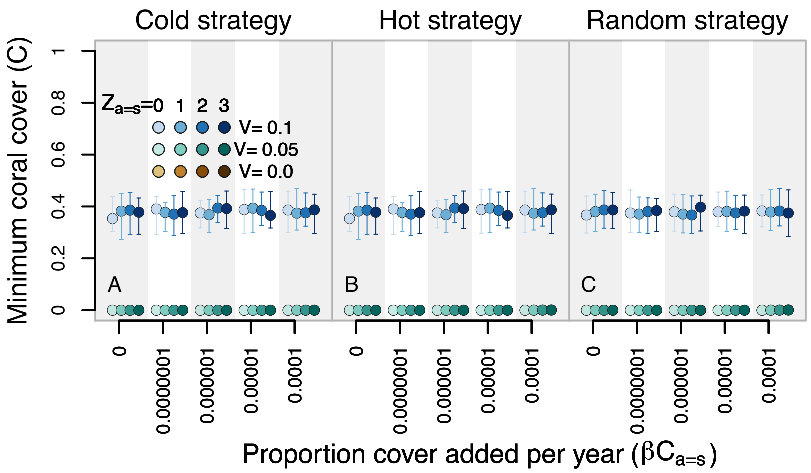

Figure S4. Effects of supplementation and assisted evolution on **minimum** coral cover. The details of this figure are identical to those of Figure S2 except that minimum coral cover is plotted on the y axis instead of mean cover at year 500. 'Minimum Coral Cover' (y axis) is the minimum amount of coral cover that occurs across the 500-year climate change scenario for a given parameter combination. These results are from simulations parameterized for alternative stable states (Table S1).

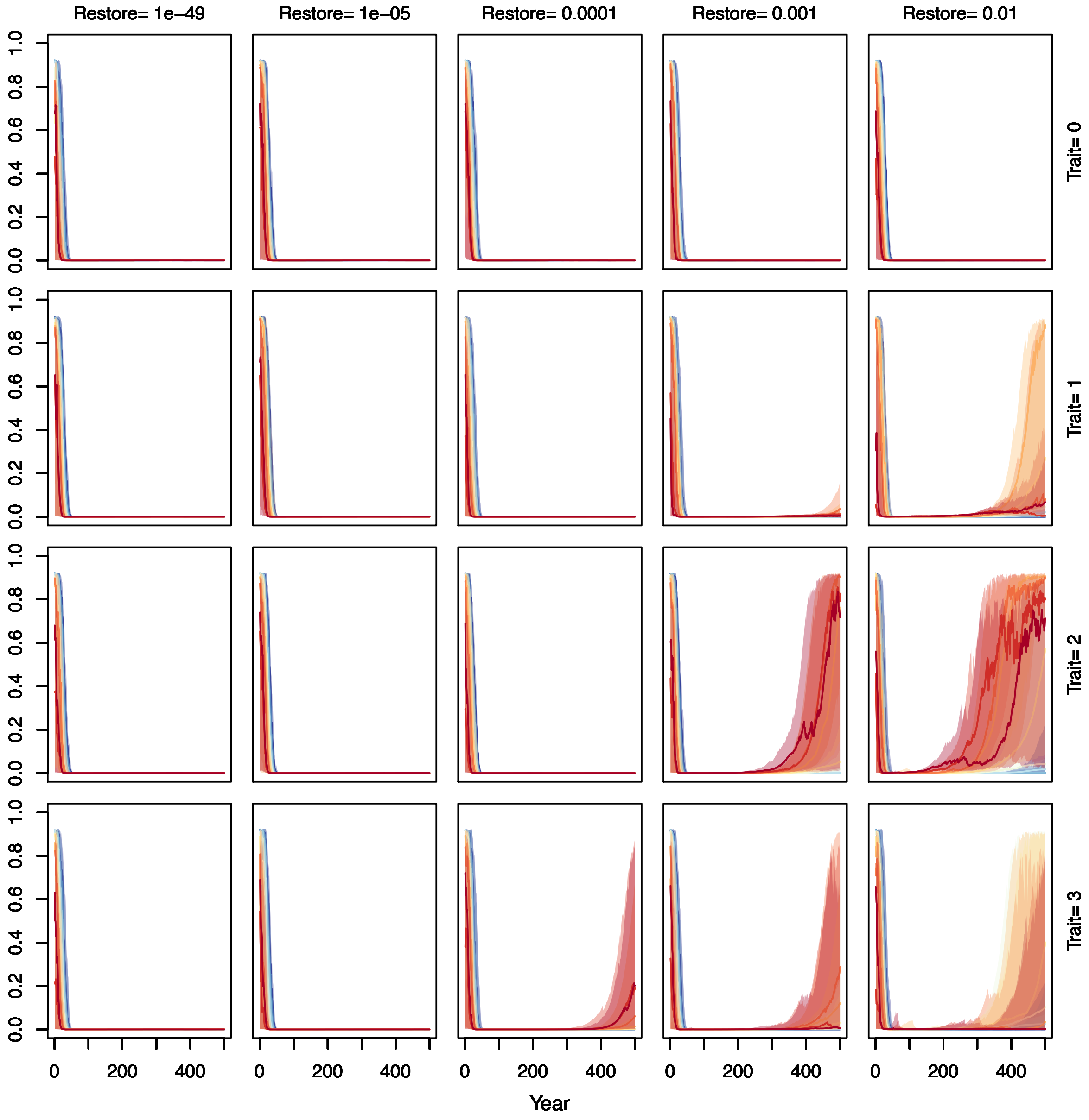

Figure S5. Simulated trajectories of individual reefs under climate change projections with varying levels of supplementation and assisted evolution. Solid lines and shaded borders represent the median and 50% quantiles (across simulations) respectively of coral cover over time at each patch within the network. Red lines represent patches exposed to higher thermal stress and blue lines show colder reefs. The y-axis shows proportional coral cover for each scenario, and the x-axis shows the year of the simulation. Column labels refer to the amount of cover at source reefs ($C_{a=s}$), which when multiplied by fecundity (β) give the effective supplementation rate ($\beta C_{a=s}$) in terms of the proportion of total benthic habitat area added to target reefs each year. Rows represent the degree of thermal trait enhancement that was applied to the corals being added to target reefs. Results here assume **no genetic variance (V=0)**, **low dispersal ($\boldsymbol{\beta = 0.01}$)**, a competition matrix parameterized for alternative stable states and a spatial strategy targeting **hot reefs**.

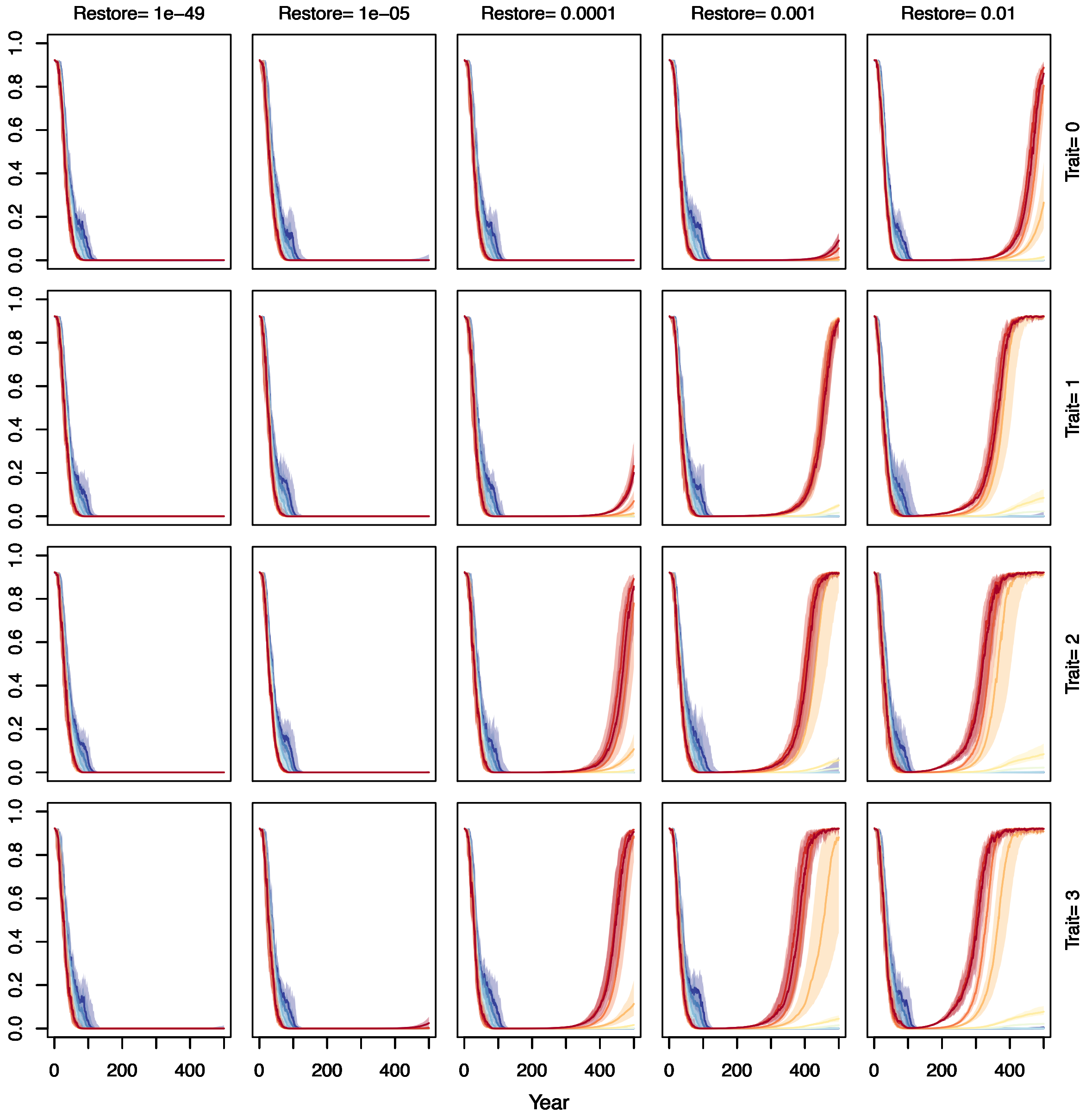

Figure S6. Simulated trajectories of individual reefs under climate change projections with varying levels of supplementation and assisted evolution. Solid lines and shaded borders represent the median and 50% quantiles (across simulations) respectively of coral cover over time at each patch within the network. Red lines represent patches exposed to higher thermal stress and blue lines show colder reefs. The y-axis shows proportional coral cover for each scenario, and the x-axis shows the year of the simulation. Column labels refer to the amount of cover at source reefs ($C_{a=s}$), which when multiplied by fecundity (β) give the effective supplementation rate ($\beta C_{a=s}$) in terms of the proportion of total benthic habitat area added to target reefs each year. Rows represent the degree of thermal trait enhancement that was applied to the corals being added to target reefs. Results here assume **low genetic variance (V=0.05)**, **low dispersal ($\boldsymbol{\beta = 0.01}$)**, a competition matrix parameterized for alternative stable states and a spatial strategy targeting **hot reefs**.

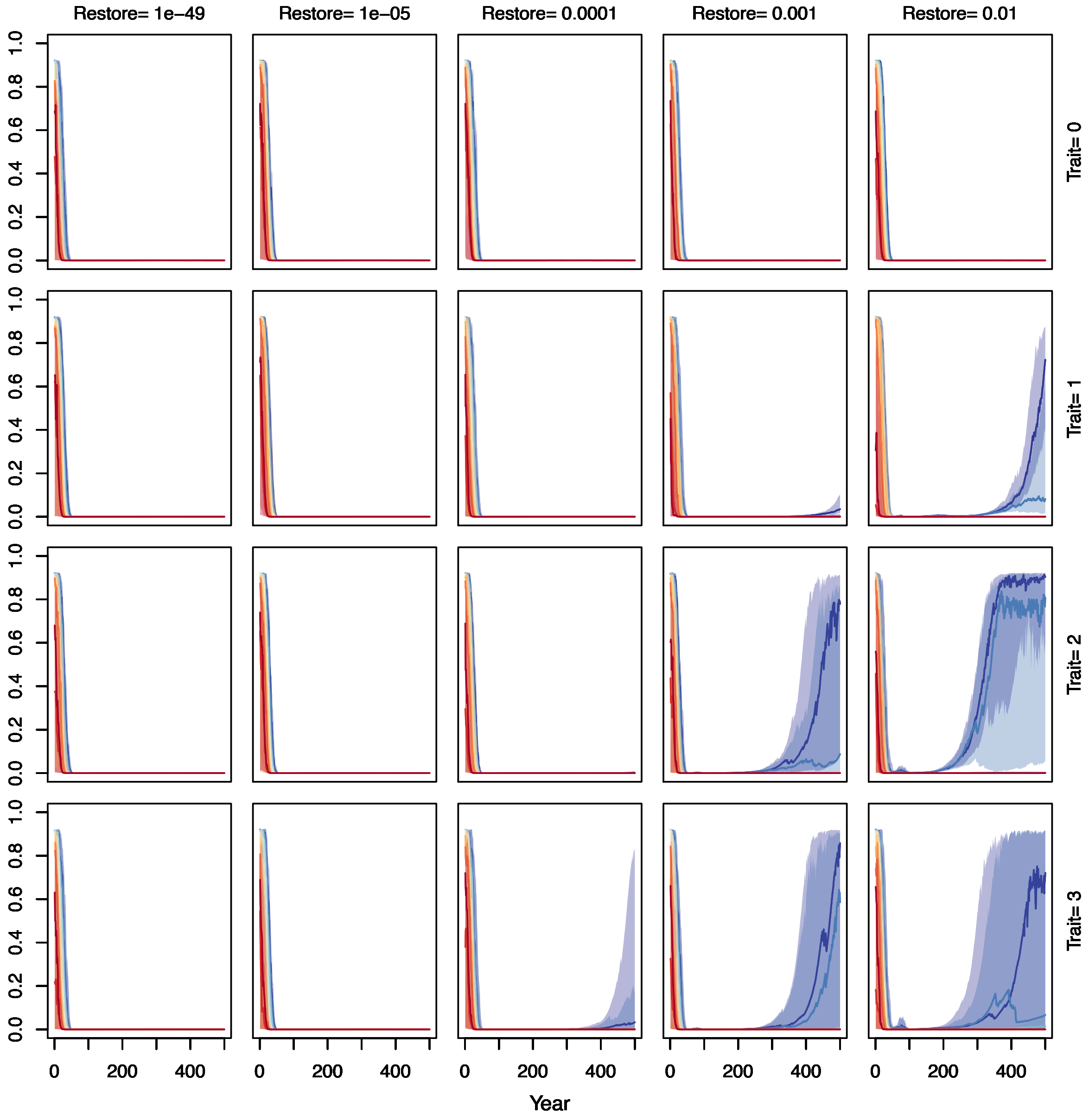

Figure S7. Simulated trajectories of individual reefs under climate change projections with varying levels of supplementation and assisted evolution. Solid lines and shaded borders represent the median and 50% quantiles (across simulations) respectively of coral cover over time at each patch within the network. Red lines represent patches exposed to higher thermal stress and blue lines show colder reefs. The y-axis shows proportional coral cover for each scenario, and the x-axis shows the year of the simulation. Column labels refer to the amount of cover at source reefs ($C_{a=s}$), which when multiplied by fecundity (β) give the effective supplementation rate ($\beta C_{a=s}$) in terms of the proportion of total benthic habitat area added to target reefs each year. Rows represent the degree of thermal trait enhancement that was applied to the corals being added to target reefs. Results here assume **no genetic variance (V=0)**, **low dispersal ($\boldsymbol{\beta = 0.01}$)**, a competition matrix parameterized for alternative stable states and a spatial strategy targeting **cold reefs**.

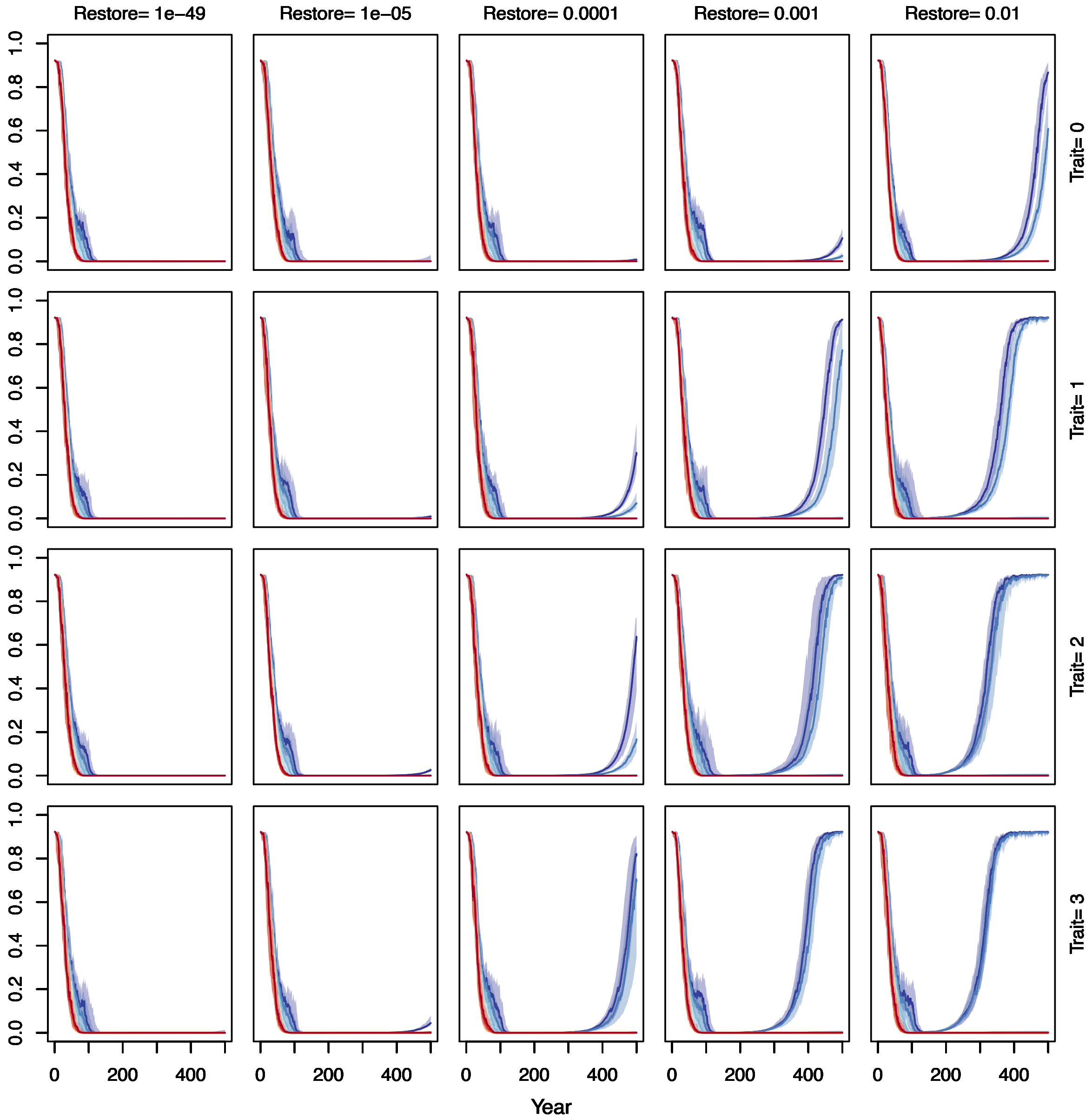

Figure S8. Simulated trajectories of individual reefs under climate change projections with varying levels of supplementation and assisted evolution. Solid lines and shaded borders represent the median and 50% quantiles (across simulations) respectively of coral cover over time at each patch within the network. Red lines represent patches exposed to higher thermal stress and blue lines show colder reefs. The y-axis shows proportional coral cover for each scenario, and the x-axis shows the year of the simulation. Column labels refer to the amount of cover at source reefs ($C_{a=s}$), which when multiplied by fecundity (β) give the effective supplementation rate ($\beta C_{a=s}$) in terms of the proportion of total benthic habitat area added to target reefs each year. Rows represent the degree of thermal trait enhancement that was applied to the corals being added to target reefs. Results here assume **low genetic variance (V=0.05)**, **low dispersal ($\boldsymbol{\beta = 0.01}$)**, a competition matrix parameterized for alternative stable states and a spatial strategy targeting **cold reefs**.

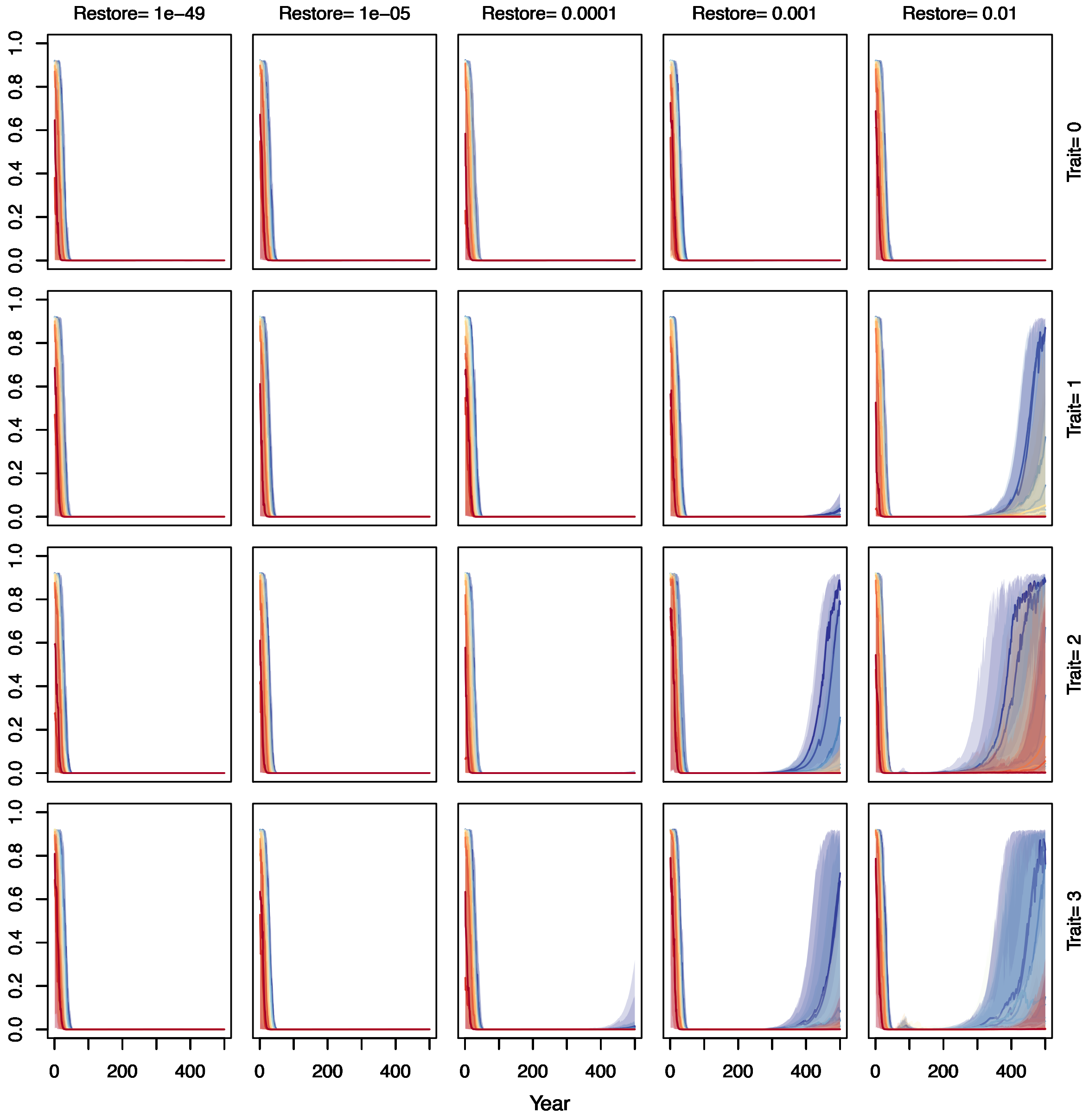

Figure S9. Simulated trajectories of individual reefs under climate change projections with varying levels of supplementation and assisted evolution. Solid lines and shaded borders represent the median and 50% quantiles (across simulations) respectively of coral cover over time at each patch within the network. Red lines represent patches exposed to higher thermal stress and blue lines show colder reefs. The y axis shows proportional coral cover for each scenario, and the y axis shows the year of the simulation. Column labels refer to the amount of cover at source reefs ($C_{a=s}$), which when multiplied by fecundity (β) give the effective supplementation rate ($\beta C_{a=s}$) in terms of the proportion of total benthic habitat area added to target reefs each year. Rows represent the degree of thermal trait enhancement that was applied to the corals being added to target reefs. Results here assume **no genetic variance (V=0)**, **low dispersal ($\boldsymbol{\beta} = \mathbf{0.01}$)**, a competition matrix parameterized for alternative stable states and a spatial strategy targeting **random reefs**.

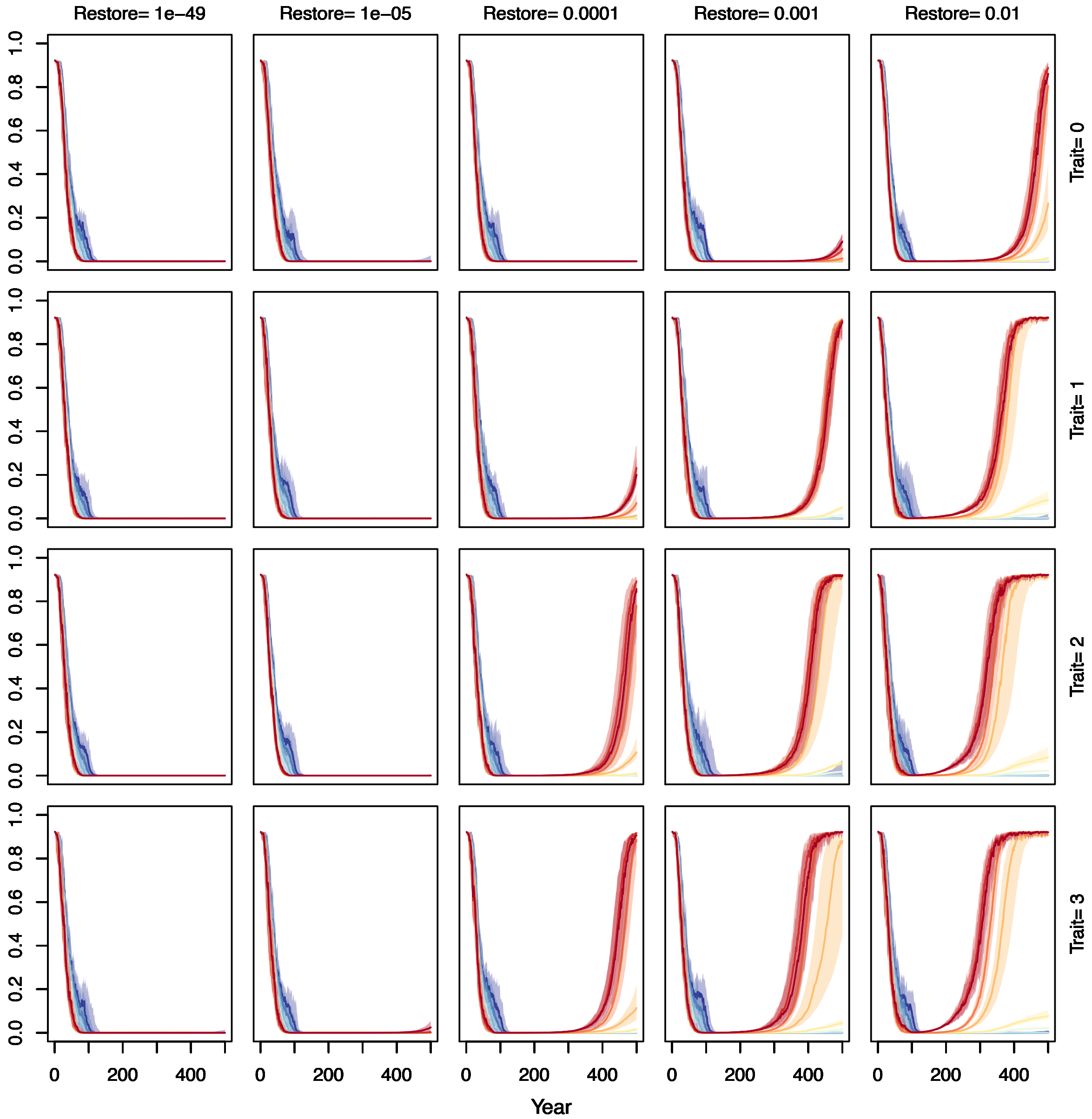

Figure S10. Simulated trajectories of individual reefs under climate change projections with varying levels of supplementation and assisted evolution. Solid lines and shaded borders represent the median and 50% quantiles (across simulations) respectively of coral cover over time at each patch within the network. Red lines represent patches exposed to higher thermal stress and blue lines show colder reefs. The y axis shows proportional coral cover for each scenario, and the y axis shows the year of the simulation. Column labels refer to the amount of cover at source reefs ($C_{a=s}$), which when multiplied by fecundity (β) give the effective supplementation rate ($\beta C_{a=s}$) in terms of the proportion of total benthic habitat area added to target reefs each year. Rows represent the degree of thermal trait enhancement that was applied to the corals being added to target reefs. Results here assume **low genetic variance (V=0.05)**, **low dispersal ($\boldsymbol{\beta = 0.01}$)**, a competition matrix parameterized for alternative stable states and a spatial strategy targeting **random reefs**.

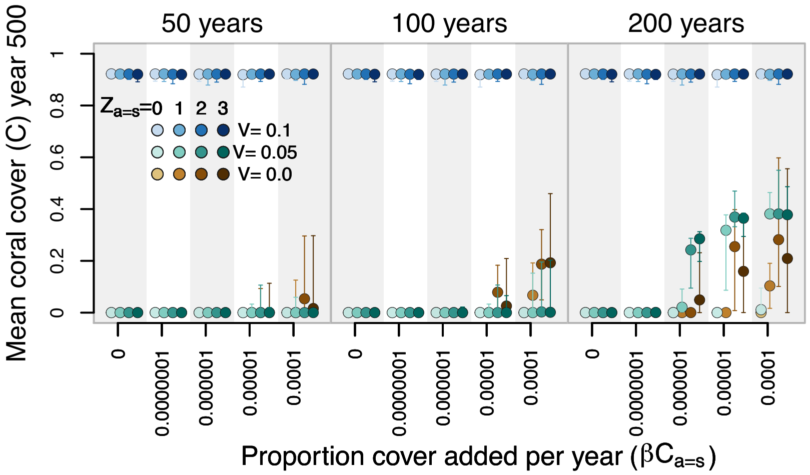

Figure S11. Effects of supplementation and assisted evolution on coral cover under alternative supplementation timelines. The details of this figure are identical to those of figure S2 except that supplementation only occurs in the first 50 (first panel), 100 (second panel), or 200 (third panel) years instead of all 500. The y axis still refers to the network-wide average at year 500 as in figure S2. These results assume a supplementation strategy targeting hot reefs and a competition matrix parameterized for alternative stable states.

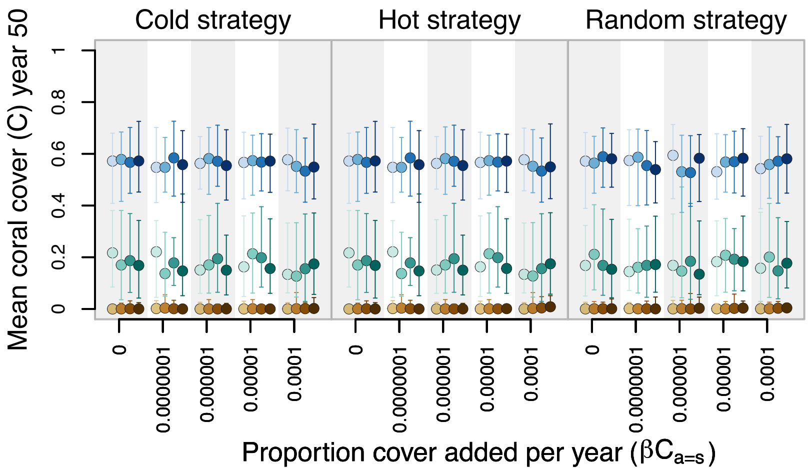

Figure S12. Effects of supplementation and assisted evolution on average coral cover across at year 50. The details of this figure are identical to those of figure S2 except that y axis shows the average across the reef network in year 50 instead of year 500 of the simulation.

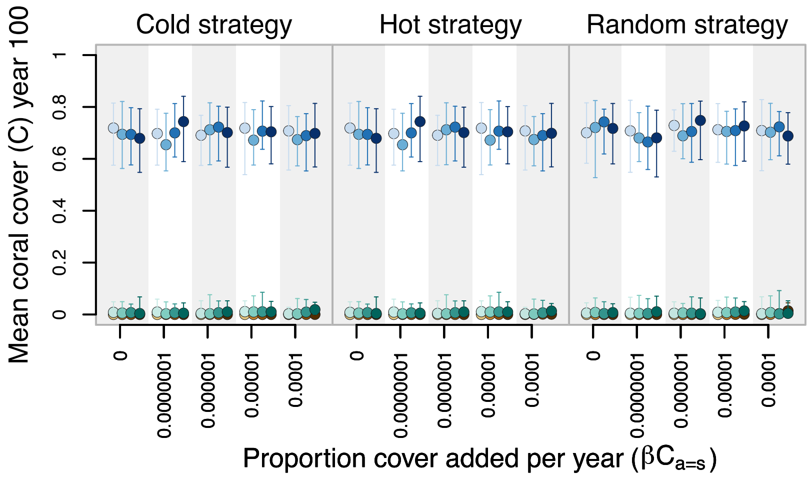

Figure S13. Effects of supplementation and assisted evolution on average coral cover across at year 100. The details of this figure are identical to those of figure S2 except that y axis shows the average across the reef network in year 100 instead of year 500 of the simulation.

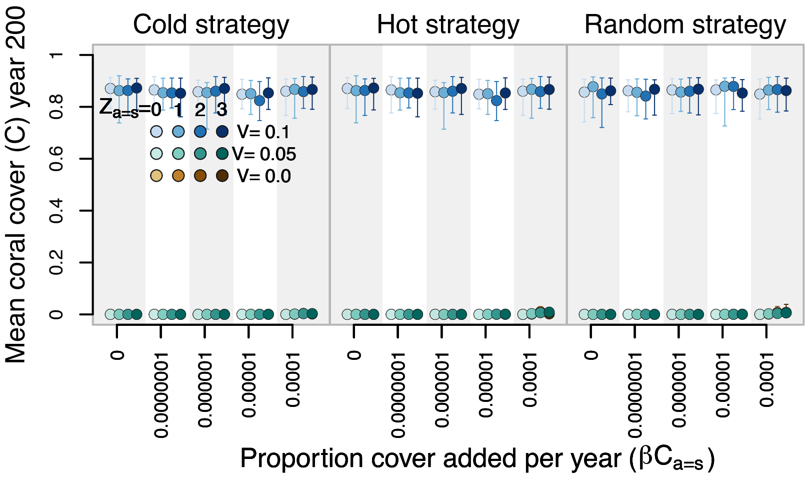

Figure S14. Effects of supplementation and assisted evolution on average coral cover across at year 200. The details of this figure are identical to those of figure S2 except that y axis shows the average across the reef network in year 200 instead of year 500 of the simulation.

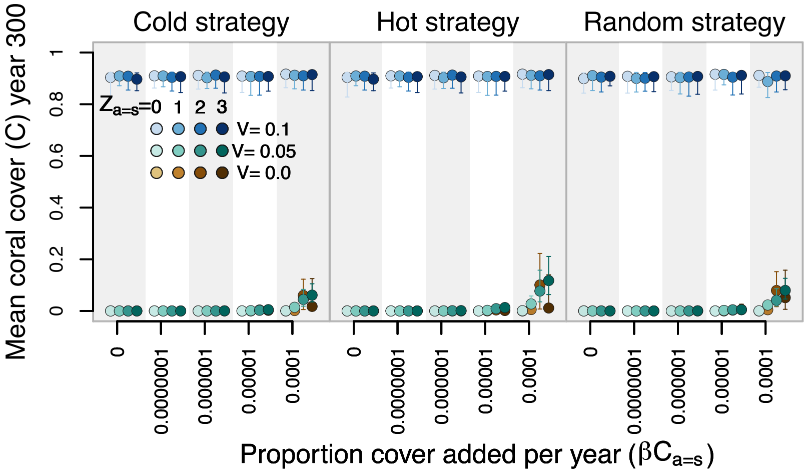

Figure S15. Effects of supplementation and assisted evolution on average coral cover across at year 300. The details of this figure are identical to those of figure S2 except that y axis shows the average across the reef network in year 300 instead of year 500 of the simulation.

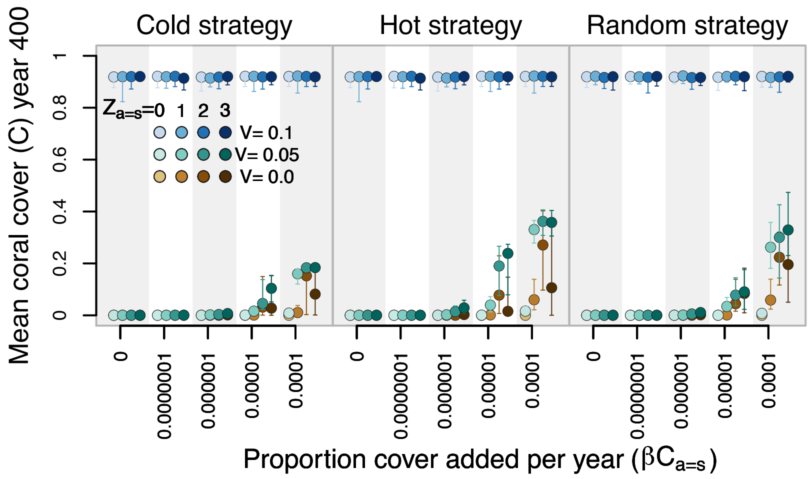

Figure S16. Effects of supplementation and assisted evolution on average coral cover across at year 400. The details of this figure are identical to those of figure S2 except that y axis shows the average across the reef network in year 400 instead of year 500 of the simulation.

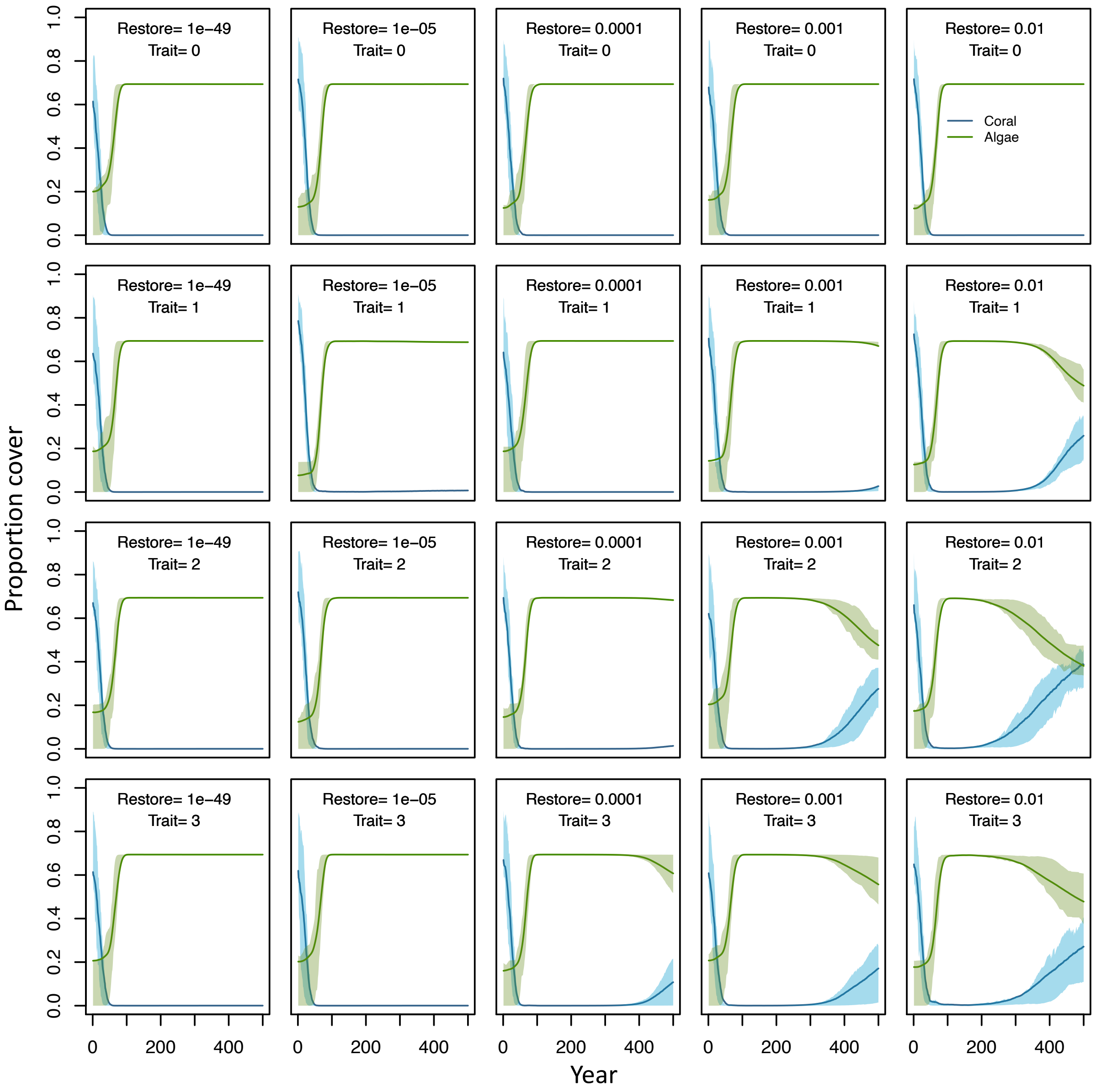

Figure S17. Relative cover of coral (blue) and macroalgae (green) across the reef network over time. Solid lines represent the median value across all simulations of the average network-wide cover, with shaded boundaries representing 50% quantiles (across simulations). Column labels (‘Restore=’) refer to the amount of cover at source reefs ($C_{a=s}$), which when multiplied by fecundity (β) give the effective supplementation rate ($\beta C_{a=s}$) in terms of the proportion of total benthic habitat area added to target reefs each year. Rows (‘Trait=’) represent the degree of thermal trait enhancement that was applied to the corals being added to target reefs. Results here assume low genetic variance (V=0.05), low dispersal ($\beta = 0.01$), a competition matrix parameterized for alternative stable states and a spatial strategy targeting hot reefs.
